# Vitamin D Deficiency Is Associated with Greater Sarcopenia-Related Risk Severity and Functional Decline in Older Adults: A Retrospective Cross-Sectional Study

**DOI:** 10.3390/medicina62081430

**Published:** 2026-07-23

**Authors:** Tulay Yildirim, Oguzhan Yildirim, Muhammed Mustafa Turker, Omer Faruk Calan, Ali Sahin, Bekir Agcakoyunlu

**Affiliations:** 1 Department of Physical Medicine and Rehabilitation, Inonu University School of Medicine, Malatya 44280, Türkiye; drtulayoner@hotmail.com (T.Y.); 592mustafaturker@gmail.com (M.M.T.); efsaneben2312@gmail.com (O.F.C.); alisahin@inonu.edu.tr (A.S.); bekirl-mbabil@hotmail.com (B.A.); 2 Department of Gastroenterology, Inonu University School of Medicine, Malatya 44280, Türkiye

**Keywords:** muscle strength, frailty, nutritional status, handgrip strength, geriatric assessment, falls

## Abstract

*Background and Objectives:* Vitamin D deficiency has been implicated in muscle weakness and functional decline among older adults. However, its association with sarcopenia-related impairment, particularly when evaluated using readily available nutritional and functional indicators in routine clinical practice, remains incompletely understood. *Materials and Methods:* This retrospective cross-sectional study included 148 adults aged ≥65 years who attended a Physical Medicine and Rehabilitation outpatient clinic. Sarcopenia-related risk was classified using an EWGSOP2-informed operational framework based on handgrip strength together with nutritional and functional indicators available in the retrospective dataset. Serum 25-hydroxyvitamin D [25(OH)D] concentrations were measured using a chemiluminescent microparticle immunoassay. Group comparisons, correlation analyses, and multivariable logistic regression analyses were performed. *Results:* The prevalence of probable sarcopenia and probable sarcopenia with nutritional/functional risk indicators was significantly higher among participants with vitamin D deficiency. Serum 25(OH)D levels differed significantly across the three study groups (*p* < 0.001). Post-hoc Tukey analysis demonstrated significantly lower serum 25(OH)D concentrations in the probable sarcopenia with nutritional/functional risk indicators group than in both the no sarcopenia and probable sarcopenia groups (both *p* < 0.001). Serum 25(OH)D levels were moderately and inversely correlated with sarcopenia-related risk severity (r = −0.48, *p* < 0.001). Lower vitamin D levels were also associated with reduced serum albumin levels and a higher prevalence of falls and assistive walking device use. In multivariable logistic regression analysis, vitamin D deficiency remained independently associated with sarcopenia-related risk after adjustment for age and sex. *Conclusions:* Vitamin D deficiency was significantly associated with greater sarcopenia-related risk severity and poorer functional status in older adults. Assessment of vitamin D status may therefore represent a simple, inexpensive, and readily available tool for identifying individuals at increased risk of sarcopenia-related impairment in routine geriatric practice. Prospective studies incorporating direct measures of muscle quantity and standardized physical performance assessments are needed to confirm these findings.

## 1. Introduction

Sarcopenia is a progressive skeletal muscle disorder characterized by age-related declines in muscle strength, muscle quantity or quality, and physical performance. It is associated with loss of functional independence, an increased risk of falls, disability, hospitalization, and mortality in older adults [1,2]. Rather than being considered solely a musculoskeletal condition, sarcopenia is now recognized as a multifactorial geriatric syndrome arising from the complex interaction of aging, nutritional deficiencies, chronic inflammation, metabolic alterations, hormonal changes, and reduced physical activity [3,4]. Consequently, its early recognition and management have become priorities across geriatrics, physical medicine and rehabilitation, and internal medicine.

Vitamin D plays an important role in skeletal muscle physiology through its effects on muscle protein synthesis, neuromuscular function, and muscle fiber maintenance, in addition to its established role in bone metabolism [5,6]. Vitamin D deficiency is highly prevalent among older adults owing to reduced sunlight exposure, inadequate dietary intake, impaired renal activation, and the increasing burden of chronic diseases [7]. Accumulating evidence indicates that low serum 25-hydroxyvitamin D [25(OH)D] concentrations are associated with reduced muscle strength, impaired physical performance, slower gait speed, and an increased risk of falls [6,7,8].

In routine clinical practice, assessment of sarcopenia frequently incorporates readily available indicators of nutritional and functional status. Low body mass index (BMI) and hypoalbuminemia have consistently been associated with malnutrition, frailty, reduced muscle reserve, and unfavorable clinical outcomes in older adults [9,10]. Likewise, recurrent falls and the need for assistive walking devices are recognized as important manifestations of declining physical function and loss of independence [11,12].

Although previous studies have demonstrated an association between vitamin D deficiency and impaired muscle strength or physical performance, relatively few have simultaneously examined its relationship with nutritional indicators and functional outcomes that are routinely available in everyday clinical practice. Consequently, the potential value of combining these easily obtainable clinical variables to characterize sarcopenia-related risk remains insufficiently explored, particularly in retrospective real-world datasets [8,13].

To our knowledge, few studies have evaluated the association between vitamin D deficiency and an operational EWGSOP2-informed classification that integrates muscle strength with routinely available nutritional and functional risk indicators. Such an approach may provide clinically useful information in settings where direct measurements of muscle quantity or physical performance are unavailable.

Therefore, the aim of the present study was to investigate the association between vitamin D deficiency and sarcopenia-related risk in older adults using an EWGSOP2-informed operational classification based on handgrip strength together with nutritional and functional indicators. We further evaluated the relationships between vitamin D status and body mass index, serum albumin levels, history of falls, and the use of assistive walking devices.

We hypothesized that vitamin D deficiency would be associated with greater sarcopenia-related risk severity, poorer nutritional status, and a higher prevalence of falls and assistive walking device use.

## 2. Materials and Methods

### 2.1. Study Design

This study was designed as a retrospective cross-sectional study to evaluate the association between serum 25-hydroxyvitamin D levels and sarcopenia-related findings in older adults. Medical records, laboratory results, and clinical assessment data of patients who attended the clinic during the study period were retrospectively reviewed. The study was reported in accordance with the Strengthening the Reporting of Observational Studies in Epidemiology (STROBE) guidelines for observational studies [14].

### 2.2. Study Population

The study population comprised adults aged ≥65 years who attended the outpatient clinics of the Department of Physical Medicine and Rehabilitation at İnönü University Faculty of Medicine during the study period. Participant selection began by restricting the electronic medical record and laboratory information system screening to individuals aged ≥65 years. All consecutive patients within this predefined age range were then assessed for eligibility according to the inclusion and exclusion criteria. The mean age of the final study population was 73.6 ± 6.8 years.

**Inclusion Criteria**

- Age ≥ 65 years (with no predefined upper age limit);
- Availability of serum 25-hydroxyvitamin D levels in clinical records;
- Documentation of muscle strength and functional status in clinical assessments;
- Recorded body mass index (BMI) and serum albumin levels;
- Availability of data regarding history of falls and/or use of assistive walking devices within the preceding year.

**Exclusion Criteria**

- Active malignancy;
- Advanced-stage chronic kidney disease;
- Acute infection or inflammatory muscle disease;
- Neuromuscular disorders;
- History of major surgery within the preceding 6 months;
- Incomplete clinical or laboratory data.

### 2.3. Definition and Classification of Sarcopenia

Sarcopenia was evaluated using an operational classification based on the recommendations of the European Working Group on Sarcopenia in Older People, second consensus report (EWGSOP2, 2019) [1]. According to EWGSOP2, sarcopenia is a progressive and generalized skeletal muscle disorder characterized by low muscle strength, reduced muscle quantity or quality, and impaired physical performance [1,2].

In accordance with the EWGSOP2 framework, low muscle strength was considered the primary criterion for identifying probable sarcopenia and was assessed using handgrip strength measurements. However, because of the retrospective design of the study, direct assessments of muscle quantity or quality, such as dual-energy X-ray absorptiometry (DXA), bioelectrical impedance analysis (BIA), computed tomography (CT), or magnetic resonance imaging (MRI), were not available.

Consequently, formal diagnosis of EWGSOP2-defined sarcopenia could not be established. Instead, patients with probable sarcopenia were further stratified according to clinically relevant nutritional and functional risk indicators, including body mass index (BMI), serum albumin level, history of falls, and use of assistive walking devices. These variables were used as surrogate indicators of nutritional status, frailty, and functional decline rather than as direct measures of muscle quantity or quality.

This operational classification was adopted to explore clinically relevant risk profiles associated with sarcopenia-related functional impairment in a real-world retrospective dataset. Therefore, the categories used in the present study should be interpreted as risk-stratification groups rather than as formal categories of EWGSOP2-defined sarcopenia.

### 2.4. Probable Sarcopenia

Muscle strength was assessed using a calibrated Jamar Hydraulic Hand Dynamometer (Patterson Medical, Warrenville, IL, USA). Handgrip strength measurements were obtained from the dominant hand, with participants seated according to standardized testing procedures. Three consecutive measurements were performed, and the highest value was used for analysis. According to the EWGSOP2 criteria, probable sarcopenia was defined as low muscle strength, with handgrip strength < 27 kg in men and <16 kg in women [1].

### 2.5. Probable Sarcopenia with Nutritional Risk Indicators

Patients with low handgrip strength who additionally had a body mass index (BMI) < 22 kg/m^2^ and/or serum albumin level < 3.5 g/dL were categorized as having probable sarcopenia with nutritional risk indicators. BMI and serum albumin were used as indicators of nutritional status rather than as direct measures of muscle quantity or quality [9,10,15,16].

### 2.6. Functional Risk Indicators

History of falls and use of assistive walking devices were evaluated as clinically relevant indicators of impaired physical performance and functional decline. Specifically, the following variables were assessed:

- History of one or more falls within the preceding year;
- Use of assistive walking devices (e.g., cane or walker).

According to EWGSOP2, advanced sarcopenia according to EWGSOP2 requires the presence of low muscle strength, low muscle quantity or quality, and impaired physical performance assessed using validated physical performance tests [1,2]. Because direct assessments of muscle quantity and standardized physical performance measures were not available in this retrospective dataset, formal advanced sarcopenia according to EWGSOP2 could not be established. Therefore, history of falls and use of assistive walking devices were incorporated as functional risk indicators reflecting impaired physical performance and reduced functional independence rather than as diagnostic criteria for advanced sarcopenia according to EWGSOP2 [4,11,12,16].

Although falls and the use of assistive walking devices are not included in the EWGSOP2 diagnostic algorithm, both variables are recognized as clinically relevant manifestations of impaired mobility, reduced physical performance, and loss of functional independence in older adults. Therefore, these indicators were incorporated solely to characterize functional impairment associated with sarcopenia-related risk and not to establish a diagnosis of advanced sarcopenia according to EWGSOP2.

### 2.7. Sarcopenia Classification Used in the Study

Patients were categorized into three groups: no sarcopenia, probable sarcopenia, and probable sarcopenia with nutritional and functional risk indicators. This operational classification was developed to facilitate risk stratification in a retrospective clinical dataset where direct assessment of muscle quantity and standardized physical performance measures were not available. The approach was informed by key EWGSOP2 concepts, particularly the central role of low muscle strength in identifying probable sarcopenia, while incorporating clinically relevant nutritional and functional risk indicators [1,2].

For analytical purposes, nutritional risk indicators (BMI < 22 kg/m^2^ and/or serum albumin < 3.5 g/dL) and functional risk indicators (history of falls and/or use of assistive walking devices) were evaluated together within the same risk-stratification category. This classification was intended to identify patients with a higher burden of sarcopenia-related nutritional and functional impairment and should not be interpreted as a formal EWGSOP2 classification.

### 2.8. Laboratory Assessment

Serum 25-hydroxyvitamin D [25(OH)D] and albumin levels were measured in the accredited central hospital laboratory using routinely applied and validated methods. All laboratory analyses were performed according to the manufacturer’s quality-control procedures and routine internal quality assurance protocols. Serum 25(OH)D concentrations were determined using a chemiluminescent microparticle immunoassay (CMIA) on the Architect i2000SR analyzer (Abbott Diagnostics, Abbott Park, IL, USA). Results were expressed as ng/mL for serum 25(OH)D and g/dL for serum albumin. According to the laboratory reference values and widely accepted clinical thresholds, vitamin D deficiency was defined as a serum 25(OH)D concentration of <20 ng/mL.

### 2.9. Clinical Assessment

Clinical data including age, sex, body mass index, history of falls, and use of assistive walking devices were obtained from electronic patient records.

### 2.10. Statistical Analysis

Statistical analyses were performed using IBM SPSS Statistics for Windows, Version 25.0 (IBM Corp., Armonk, NY, USA). The distribution of continuous variables was assessed using the Kolmogorov–Smirnov test. Normally distributed data are presented as mean ± standard deviation, whereas non-normally distributed data are presented as median (minimum–maximum).

Continuous variables with a normal distribution were compared using one-way analysis of variance (ANOVA), whereas non-normally distributed variables were analyzed using the Kruskal–Wallis test. When the overall ANOVA result was statistically significant, pairwise comparisons were performed using Tukey’s post-hoc test. For one-way ANOVA, effect sizes were estimated using eta squared (η^2^). Categorical variables were compared using the chi-square test.

For correlation analyses, sarcopenia-related risk categories were coded as ordinal numerical values: 0 = no sarcopenia, 1 = probable sarcopenia, and 2 = probable sarcopenia with nutritional and functional risk indicators. Pearson correlation analysis was subsequently performed using these coded values.

Multivariable logistic regression analysis was conducted to identify factors independently associated with the presence of sarcopenia-related risk. Age, sex, body mass index, serum albumin level, and serum 25-hydroxyvitamin D levels were included in the regression model. Because BMI and serum albumin constituted components of the operational risk-stratification framework rather than direct measures of muscle quantity, these variables were retained in the model to evaluate their relative contribution to sarcopenia-related risk. The results should therefore be interpreted as associations within the operational classification adopted in this study rather than as determinants of formally defined EWGSOP2 sarcopenia. Odds ratios (ORs) with 95% confidence intervals (CIs) were reported for logistic regression analyses.

A two-sided *p* value < 0.05 was considered statistically significant.

### 2.11. Bias Control

To reduce selection bias, all consecutive patients meeting the predefined inclusion and exclusion criteria during the study period were included in the analysis. Standardized laboratory procedures and routinely recorded clinical assessments were used to minimize information bias. Potential confounding factors were addressed through multivariable logistic regression analysis adjusted for age and sex. Nevertheless, because of the retrospective design of the study, residual confounding and unmeasured bias cannot be completely excluded.

### 2.12. Ethical Approval

The study protocol was approved by the Inonu University Clinical Research Ethics Committee (Decision No. 2026/9265). Due to the retrospective nature of the study, the requirement for written informed consent was waived by the ethics committee. All data were anonymized prior to analysis, and the study was conducted in accordance with the principles of the Declaration of Helsinki.

## 3. Results

A total of 148 older adults who met the inclusion criteria were retrospectively evaluated. The baseline demographic, clinical, and laboratory characteristics of the study population are summarized in Table 1. Corresponding characteristics stratified by sex and age group (65–74 years vs. ≥75 years) are provided in Supplementary Tables S1 and S2. The mean age was 73.6 ± 6.8 years; 92 patients (62.2%) were women and 56 (37.8%) were men. The mean body mass index (BMI) was 24.1 ± 3.9 kg/m^2^, the mean serum albumin level was 3.6 ± 0.5 g/dL, and the mean serum 25-hydroxyvitamin D [25(OH)D] level was 17.9 ± 7.6 ng/mL. Sixty-four patients (43.2%) reported at least one fall within the preceding year, and 52 patients (35.1%) were using an assistive walking device (cane or walker).

### 3.1. Distribution According to Sarcopenia Classification

According to the operational sarcopenia-related risk classification used in this study, 54 participants (36.5%) had no sarcopenia, 46 (31.1%) had probable sarcopenia, and 48 (32.4%) were classified as having probable sarcopenia with nutritional and functional risk indicators. With increasing sarcopenia-related risk severity, serum 25(OH)D and albumin levels decreased significantly, whereas the prevalence of a history of falls and use of assistive walking devices increased (*p* < 0.001).

### 3.2. Association Between Vitamin D Levels and Sarcopenia

Serum 25(OH)D levels showed a significant decreasing trend across sarcopenia groups (one-way ANOVA, *p* < 0.001). Post-hoc Tukey analysis demonstrated that the Probable Sarcopenia with Nutritional/Functional Risk Indicators group had significantly lower serum 25(OH)D levels than both the No Sarcopenia and Probable Sarcopenia groups (both *p* < 0.001). Similarly, BMI and serum albumin levels decreased significantly with increasing sarcopenia-related risk severity (*p* < 0.01; Table 2).

### 3.3. History of Falls and Use of Assistive Walking Devices

Patients with a history of falls had significantly lower serum 25(OH)D levels compared with those without falls (15.2 ± 6.4 vs. 20.1 ± 7.8 ng/mL; *p* = 0.001). Likewise, serum 25(OH)D levels were significantly lower in patients using assistive walking devices than in those who did not (14.6 ± 6.1 vs. 21.0 ± 7.5 ng/mL; *p* < 0.001).

Within the operational sarcopenia-related risk classification, the probable sarcopenia with nutritional and functional risk indicators group exhibited significantly higher rates of a history of falls (62.5%) and assistive walking device use (58.3%) than the other groups (*p* < 0.001) (Table 3).

### 3.4. Multivariable Analysis

In multivariable logistic regression analysis evaluating factors associated with the presence of sarcopenia-related risk vitamin D deficiency, serum albumin < 3.5 g/dL, and BMI < 22 kg/m^2^ were independently associated with higher sarcopenia-related risk (Table 4). These associations remained significant after adjustment for age and sex.

To evaluate the robustness of the findings, a sensitivity analysis excluding BMI and serum albumin from the regression model was performed. Vitamin D deficiency remained independently associated with higher sarcopenia-related risk, indicating that the principal findings were not materially altered. However, the regression coefficients for BMI and serum albumin in Model 1 should be interpreted cautiously because these variables were also components of the operational risk classification, resulting in partial circularity and a potential overestimation of their associations.

### 3.5. Correlation Analysis

**Figure 1:**
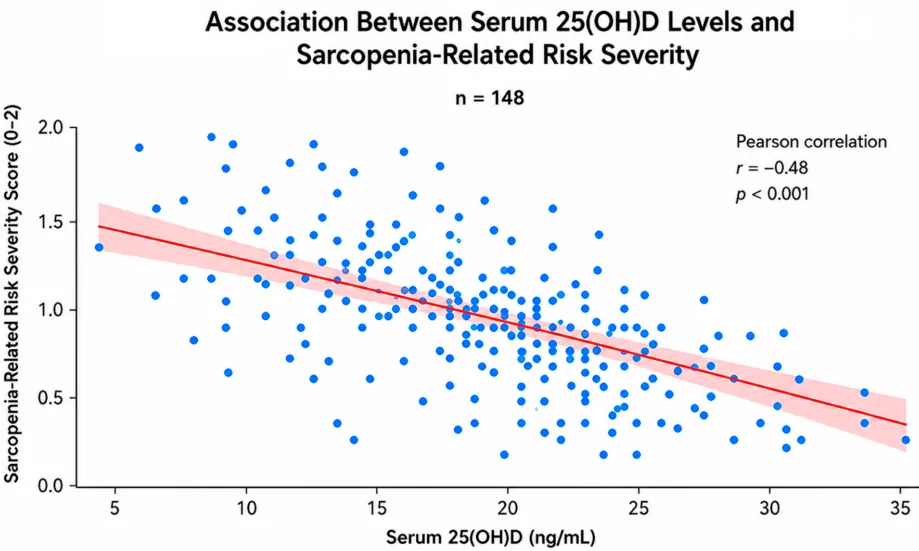
Scatter plot showing the association between serum 25-hydroxyvitamin D [25(OH)D] levels and sarcopenia-related risk severity in older adults (*n* = 148). Pearson correlation analysis demonstrated a moderate negative correlation (*r* = −0.48, *p* < 0.001). The red line represents the linear regression line and the shaded area indicates the 95% confidence interval.

Pearson correlation analysis demonstrated a moderate negative correlation between serum 25(OH)D levels and sarcopenia-related risk severity (r = −0.48, *p* < 0.001). In addition, serum 25(OH)D levels were positively correlated with serum albumin levels (r = 0.41, *p* < 0.001). These findings indicate that lower vitamin D levels were associated with greater sarcopenia-related risk severity (Figure 1).

### 3.6. Vitamin D Levels According to Assistive Device Use

**Figure 2:**
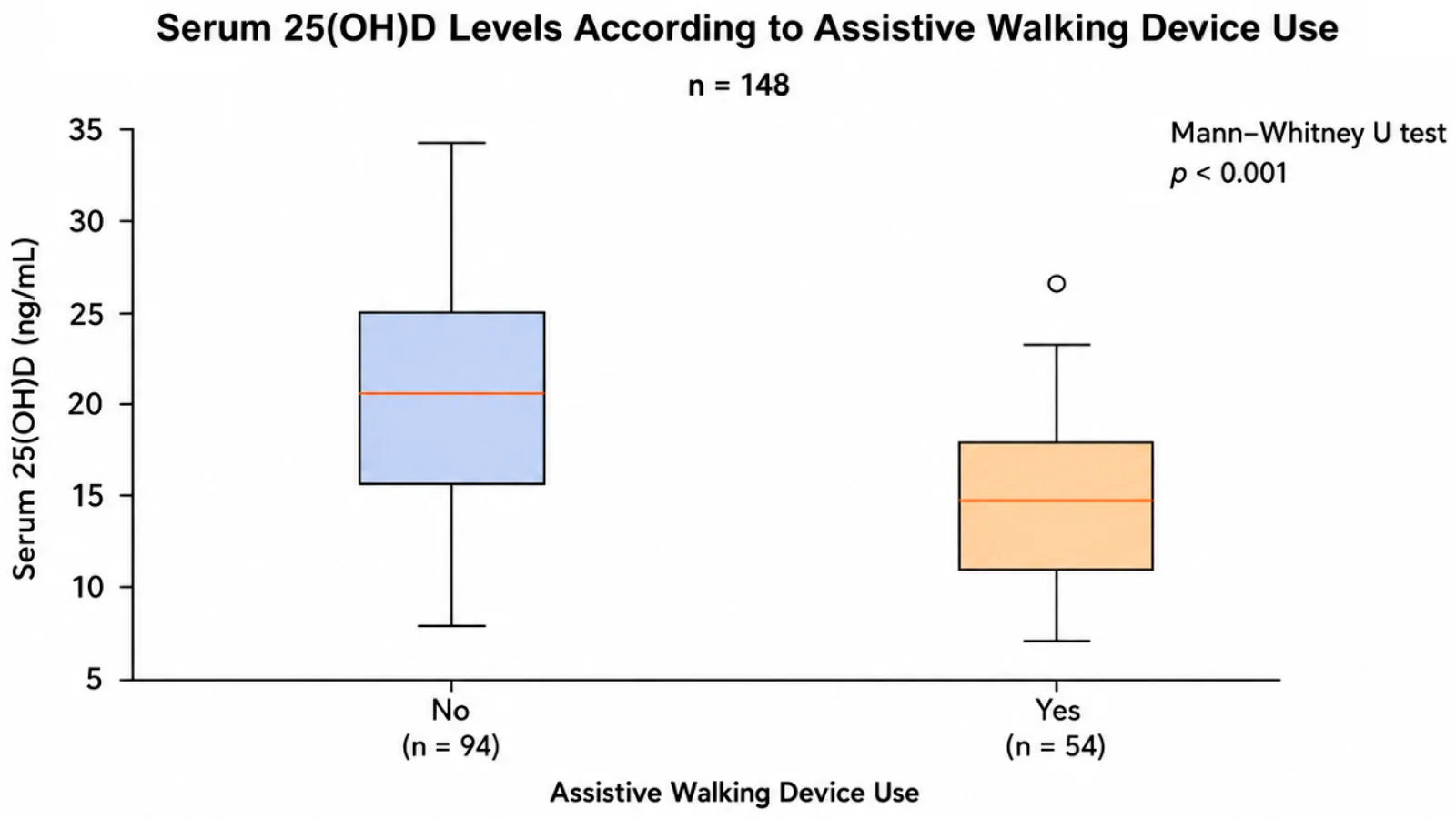
Box plot depicting the distribution of serum 25(OH)D levels according to assistive walking device use. Patients using assistive walking devices (Yes) had significantly lower serum 25(OH)D levels compared with those who did not use assistive devices (No) (*p* < 0.001). The box represents the interquartile range (IQR), the horizontal line indicates the median, and circles represent outliers.

The median serum 25(OH)D level was significantly lower in patients using assistive walking devices compared with those who did not (13.8 ng/mL [8.4–22.6] vs. 20.5 ng/mL [11.2–34.1]; *p* < 0.001) (Figure 2).

## 4. Discussion

The present study evaluated the association between serum 25-hydroxyvitamin D [25(OH)D] concentrations and sarcopenia-related risk in older adults using an EWGSOP2-informed operational classification. The principal finding was that vitamin D deficiency was associated with a greater burden of nutritional and functional impairment among individuals with probable sarcopenia. Participants with deficient vitamin D levels also exhibited lower serum albumin concentrations, lower body mass index (BMI), a higher prevalence of falls, and more frequent use of assistive walking devices. Importantly, these associations remained significant after adjustment for age and sex.

Sarcopenia is increasingly recognized as a complex geriatric syndrome involving impaired muscle strength, declining physical performance, and increased susceptibility to disability, hospitalization, and mortality [1,2,3]. The EWGSOP2 consensus identifies low muscle strength as the primary criterion for identifying probable sarcopenia and recommends direct assessment of muscle quantity and physical performance for confirmation and staging [2]. Consistent with these recommendations, handgrip strength served as the principal indicator of muscle function in the present study.

Because of the retrospective study design, direct assessments of muscle quantity or quality using dual-energy X-ray absorptiometry (DXA), bioelectrical impedance analysis (BIA), computed tomography (CT), or magnetic resonance imaging (MRI) were unavailable. Consequently, formal EWGSOP2-defined sarcopenia could not be established. Instead, nutritional and functional indicators were incorporated into an operational risk-stratification framework. Although this approach does not replace standard diagnostic criteria, it provides a pragmatic method for evaluating patients in real-world settings where advanced body composition measurements are unavailable.

The observed association between vitamin D deficiency and greater sarcopenia-related risk is biologically plausible. Vitamin D receptors are expressed in skeletal muscle tissue, and experimental studies have demonstrated that vitamin D influences muscle protein synthesis, muscle fiber composition, neuromuscular transmission, and muscle regeneration [5,6,13]. Deficiency has been associated with type II muscle fiber atrophy, reduced muscle strength, impaired balance, and diminished physical performance, all of which may contribute to functional decline in aging populations [6,13].

Our findings are consistent with previous observational studies reporting associations between lower serum 25(OH)D concentrations and reduced muscle strength, poorer physical performance, and increased fall risk [6,7,8]. Houston et al. demonstrated that lower vitamin D concentrations were associated with impaired physical function [8], whereas Bischoff-Ferrari et al. reported better muscle strength, balance, and fewer falls among individuals with adequate vitamin D status [6]. Similarly, participants with vitamin D deficiency in the present study experienced falls more frequently and were more likely to require assistive walking devices, suggesting reduced functional independence.

Recent systematic reviews and meta-analyses have further strengthened the evidence linking vitamin D deficiency with impaired muscle function, sarcopenia, and adverse functional outcomes [17,18,19]. Although the effects of vitamin D supplementation remain heterogeneous across studies, correction of deficiency appears to improve muscle strength and physical performance, particularly in individuals with low baseline vitamin D concentrations [17,18]. In addition, prospective cohort studies indicate that lower baseline serum 25(OH)D levels are associated with a greater risk of incident sarcopenia and subsequent functional decline [19]. Collectively, these findings support routine assessment of vitamin D status as part of a comprehensive geriatric evaluation.

More recently, additional systematic reviews and meta-analyses published in 2024 and 2025 have provided further evidence supporting the association between vitamin D status and sarcopenia-related outcomes. A systematic review and meta-analysis including more than 6600 older adults reported that vitamin D supplementation was associated with modest improvements in muscle strength and physical performance, whereas its effects on muscle mass remained inconsistent. The greatest benefits were observed among individuals with baseline vitamin D deficiency, suggesting that correction of deficiency may be particularly relevant in this subgroup [20]. Furthermore, a recent systematic review and meta-analysis demonstrated a significant inverse association between serum 25-hydroxyvitamin D concentrations and frailty, supporting vitamin D status as a practical biomarker for identifying older adults at increased risk of adverse functional outcomes [21]. Because frailty, sarcopenia, falls, and functional decline frequently coexist in geriatric populations, these findings further reinforce the clinical relevance of routine vitamin D assessment as part of comprehensive geriatric evaluation. In addition, a recent narrative review emphasized that although observational evidence consistently links vitamin D deficiency with impaired muscle strength and sarcopenia, the heterogeneity of randomized controlled trials suggests that vitamin D supplementation should primarily be targeted to individuals with documented deficiency rather than universally recommended [22]. These contemporary findings are in agreement with the present results, in which vitamin D deficiency remained independently associated with greater sarcopenia-related risk after adjustment for age and sex.

Markers of nutritional status also demonstrated important associations with the operational risk classification. Lower serum albumin concentrations and lower BMI were consistently observed among participants with greater nutritional and functional impairment. Low BMI and hypoalbuminemia have been associated with malnutrition, frailty, reduced muscle reserve, functional decline, and adverse clinical outcomes in older adults [9,10,15]. Although these variables are not direct measures of muscle quantity and are not included as diagnostic criteria in the EWGSOP2 algorithm, they may provide clinically relevant information regarding nutritional vulnerability in older adults at risk of sarcopenia [16]. Accordingly, in the present study, BMI and serum albumin were used solely as nutritional risk indicators within the operational classification and should not be interpreted as substitutes for direct assessment of muscle quantity or quality.

Likewise, the higher prevalence of falls and assistive walking device use among participants with greater sarcopenia-related risk emphasizes the functional consequences of impaired muscle strength. These observations are consistent with previous studies identifying muscle weakness as an important determinant of falls, disability, and loss of independence in aging populations [4,11,12]. Together, the present findings highlight the potential value of integrating nutritional and functional information when evaluating older adults at increased risk of sarcopenia.

Despite these findings, the cross-sectional design precludes conclusions regarding causality. Whether vitamin D deficiency directly contributes to the development of sarcopenia-related impairment or instead represents a marker of poorer overall health status cannot be determined from the present data. Prospective longitudinal studies incorporating direct measurements of muscle quantity, standardized physical performance assessments, and randomized controlled trials evaluating vitamin D supplementation are needed to clarify the causal nature of these associations. Furthermore, the persistence of the association between vitamin D deficiency and sarcopenia-related risk in the sensitivity analysis supports the robustness of the findings despite the operational classification adopted in the present study.

### 4.1. Strengths of the Study

The present study has several strengths. First, it evaluated the association between vitamin D status and sarcopenia-related risk using a relatively large real-world cohort of older adults assessed in a routine outpatient setting, thereby enhancing the practical applicability of the findings. Second, the study adopted an EWGSOP2-informed operational classification, enabling systematic risk stratification based on handgrip strength together with routinely available nutritional and functional indicators in a retrospective dataset. Third, in addition to muscle strength, the analysis incorporated clinically meaningful functional outcomes, including history of falls and the use of assistive walking devices. These measures provide complementary information regarding functional decline beyond conventional laboratory and anthropometric parameters. Finally, multivariable logistic regression analysis demonstrated that the association between vitamin D deficiency and sarcopenia-related risk remained significant after adjustment for age and sex, supporting the robustness of the observed associations.

From a practical perspective, the present findings highlight the potential value of routine vitamin D assessment in older adults presenting with reduced muscle strength or functional decline. Because serum 25(OH)D measurement is inexpensive, widely available, and easily incorporated into everyday clinical care, identification of vitamin D deficiency may help recognize individuals at increased risk of sarcopenia-related impairment. Although causality cannot be inferred from this study, incorporating vitamin D assessment into comprehensive geriatric evaluation may facilitate earlier risk identification and support multidisciplinary strategies aimed at preserving muscle function and functional independence.

### 4.2. Limitations

Several limitations should be considered when interpreting the present findings. First, the retrospective cross-sectional design precludes any inference regarding causal relationships between vitamin D deficiency and sarcopenia-related impairment. Second, direct assessments of muscle quantity and quality, including dual-energy X-ray absorptiometry (DXA), bioelectrical impedance analysis (BIA), computed tomography (CT), and magnetic resonance imaging (MRI), were unavailable. Consequently, formal EWGSOP2-defined sarcopenia could not be established. Instead, handgrip strength together with routinely recorded nutritional and functional indicators—including body mass index, serum albumin levels, history of falls, and assistive walking device use—were incorporated into an operational risk-stratification approach. Although this pragmatic strategy reflects everyday clinical care and enabled evaluation of patients in a real-world setting, it may have introduced misclassification bias and influenced the estimated prevalence of individuals classified as having greater sarcopenia-related risk. Because several nutritional and functional variables were components of the operational risk-stratification framework, the observed associations involving these variables should be interpreted cautiously, as partial circularity may have resulted in overestimation of some effect estimates.

Third, several potentially important confounding variables could not be comprehensively assessed. These included physical activity, nutritional and protein intake, vitamin D supplementation, osteoporosis treatment, cognitive function, frailty status, seasonal variation in serum vitamin D measurements, and comorbid conditions that may influence both vitamin D status and muscle function. Accordingly, residual confounding cannot be excluded and may have affected the observed associations.

In addition, an a priori sample size calculation was not performed because all eligible patients meeting the predefined inclusion and exclusion criteria during the study period were included. Consequently, the study may have been underpowered to detect modest associations.

Finally, the relatively high prevalence of sarcopenia-related risk may partly reflect referral bias because participants were recruited from a tertiary-care Physical Medicine and Rehabilitation outpatient clinic. Individuals attending such clinics are more likely to have mobility limitations, musculoskeletal disorders, functional impairment, and nutritional problems than community-dwelling older adults. Therefore, these findings should be generalized with caution. Future prospective multicenter studies incorporating direct body composition measurements together with standardized assessments of physical performance are needed to validate these findings and further clarify the relationship between vitamin D deficiency and sarcopenia-related impairment.

## 5. Conclusions

The present study demonstrated that lower serum 25-hydroxyvitamin D [25(OH)D] concentrations were associated with greater sarcopenia-related risk severity in older adults. Using an EWGSOP2-informed operational classification, participants with vitamin D deficiency were more likely to exhibit probable sarcopenia together with nutritional and functional risk indicators, independent of age and sex. Importantly, these findings apply to the operational classification of sarcopenia-related risk used in the present study and should not be interpreted as evidence regarding formally diagnosed EWGSOP2-defined sarcopenia.

These findings support the potential value of routine vitamin D assessment as part of comprehensive geriatric evaluation, particularly in individuals presenting with reduced muscle strength or functional impairment. Because serum 25(OH)D measurement is inexpensive, widely available, and easily incorporated into everyday clinical care, identification of deficiency may facilitate recognition of individuals at increased risk of sarcopenia-related impairment and guide appropriate nutritional and multidisciplinary interventions.

Given the observational nature of this study, no causal relationship can be inferred between vitamin D deficiency and sarcopenia-related outcomes. Prospective longitudinal studies incorporating direct measurements of muscle quantity, standardized physical performance assessments, and randomized controlled trials of vitamin D supplementation are warranted to determine whether correction of deficiency influences sarcopenia progression and functional outcomes.

## Figures and Tables

**Table 1:** Baseline demographic, clinical, and laboratory characteristics of the study population (*n* = 148).

Variable	Value
Age (years)	73.6 ± 6.8
Sex (Female/Male)	92 (62.2%)/56 (37.8%)
BMI (kg/m^2^)	24.1 ± 3.9
Serum 25(OH)D (ng/mL)	17.9 ± 7.6
Vitamin D deficiency (<20 ng/mL)	96 (64.9%)
Serum albumin (g/dL)	3.6 ± 0.5
History of falls	64 (43.2%)
Use of assistive walking device	52 (35.1%)

Values are presented as mean ± standard deviation or number (%). Abbreviations: BMI, body mass index; 25(OH)D, 25-hydroxyvitamin D.

**Table 2:** Serum 25(OH)D, BMI, and serum albumin levels according to sarcopenia-related risk groups (*n* = 148).

Variable	No Sarcopenia (*n* = 54)	Probable Sarcopenia (*n* = 46)	Probable Sarcopenia with Nutritional/Functional Risk Indicators (*n* = 48)	η^2^	*p* Value
Serum 25(OH)D (ng/mL)	23.1 ± 7.2	18.6 ± 6.8	13.4 ± 5.9	0.27	<0.001
BMI (kg/m^2^)	26.1 ± 3.4	24.3 ± 3.1	21.8 ± 2.9	0.25	<0.01
Serum albumin (g/dL)	3.9 ± 0.4	3.6 ± 0.4	3.2 ± 0.5	0.31	<0.01

Values are presented as mean ± standard deviation. Group comparisons were performed using one-way analysis of variance (ANOVA). Effect sizes are presented as eta squared (η^2^). Abbreviations: BMI, body mass index; 25(OH)D, 25-hydroxyvitamin D.

**Table 3:** Distribution of history of falls and assistive walking device use according to sarcopenia-related risk group.

Sarcopenia-Related Risk Group	History of Falls, n (%)	Assistive Walking Device Use, n (%)
No Sarcopenia	12 (22.2%)	8 (14.8%)
Probable Sarcopenia	22 (47.8%)	16 (34.8%)
Probable Sarcopenia with Nutritional/Functional Risk Indicators	30 (62.5%)	28 (58.3%)

Values are presented as number (%). Group comparisons were performed using the χ^2^ test.

**Table 4:** Multivariable logistic regression analysis of factors associated with higher sarcopenia-related risk.

Variable	Model 1 (Original) OR (95% CI)	*p* Value	Model 2 (BMI and Albumin Excluded) OR (95% CI)	*p* Value
Vitamin D deficiency	2.87 (1.42–5.79)	0.003	2.31 (1.18–4.51)	0.015
Age	1.03 (0.99–1.08)	0.18	1.02 (0.99–1.05)	0.23
Female sex	1.09 (0.65–1.83)	0.74	1.12 (0.64–1.96)	0.69
BMI < 22 kg/m^2^	2.46 (1.17–5.17)	0.018	—	—
Serum albumin < 3.5 g/dL	3.21 (1.42–7.26)	0.005	—	—

Note: Model 2 was constructed as a sensitivity analysis after excluding BMI and serum albumin because these variables were components of the operational sarcopenia-related risk classification. Abbreviations: BMI, body mass index; CI, confidence interval; OR, odds ratio.

## Data Availability

The data that support the findings of this study are available from the corresponding author upon reasonable request.

## Supplementary Materials

The following supporting information can be downloaded at: https://www.mdpi.com/article/10.3390/medicina62081430/s1, Table S1: Baseline demographic, clinical, and laboratory characteristics according to sex; Table S2: Baseline demographic, clinical, and laboratory characteristics according to age group.
