# Supplementary material for: Vitamin D Deficiency Is Associated with Greater Sarcopenia-Related Risk Severity and Functional Decline in Older Adults: A Retrospective Cross-Sectional Study
Source: Medicina (Kaunas). 2026 Jul 23;62(8):1430. doi: 10.3390/medicina62081430 (PMC13514520; doi:10.3390/medicina62081430)
Supplement: Supplementary file 1 [file medicina-62-01430-s001.zip › medicina-4284726-supplementary.pdf]

**Supplementary Table S1. Baseline demographic, clinical, and laboratory characteristics according to sex.**

| Variable | Male (n = 56) | Female (n = 92) | p value |
|---|---|---|---|
| Age (years) | 72.8 ± 6.5 | 74.1 ± 6.9 | 0.28 |
| BMI (kg/m²) | 24.5 ± 3.8 | 23.9 ± 4.0 | 0.39 |
| Serum 25(OH)D (ng/mL) | 18.6 ± 7.5 | 17.5 ± 7.7 | 0.44 |
| Vitamin D deficiency (<20 ng/mL) | 34 (60.7%) | 62 (67.4%) | 0.41 |
| Serum albumin (g/dL) | 3.7 ± 0.5 | 3.6 ± 0.5 | 0.31 |
| History of falls | 21 (37.5%) | 43 (46.7%) | 0.29 |
| Use of assistive walking device | 17 (30.4%) | 35 (38.0%) | 0.35 |

*Values are presented as mean ± standard deviation or number (%). Continuous variables were compared using the independent-samples t-test, and categorical variables were compared using the $\chi^2$ test, as appropriate. Abbreviations: BMI, body mass index; 25(OH)D, 25-hydroxyvitamin D.*

**Supplementary Table S2. Baseline demographic, clinical, and laboratory characteristics according to age group.**

| Variable | 65–74 years (n = 79) | ≥75 years (n = 69) | p value |
|---|---|---|---|
| Female sex | 47 (59.5%) | 45 (65.2%) | 0.48 |
| BMI (kg/m²) | 24.7 ± 3.6 | 23.4 ± 4.1 | 0.06 |
| Serum 25(OH)D (ng/mL) | 18.8 ± 7.3 | 16.8 ± 7.8 | 0.11 |
| Vitamin D deficiency (<20 ng/mL) | 47 (59.5%) | 49 (71.0%) | 0.14 |
| Serum albumin (g/dL) | 3.7 ± 0.4 | 3.5 ± 0.5 | 0.04 |
| History of falls | 28 (35.4%) | 36 (52.2%) | 0.04 |
| Use of assistive walking device | 23 (29.1%) | 29 (42.0%) | 0.09 |

*Values are presented as mean ± standard deviation or number (%). Continuous variables were compared using the independent-samples t-test, and categorical variables were compared using the $\chi^2$ test, as appropriate. Abbreviations: BMI, body mass index; 25(OH)D, 25-hydroxyvitamin D.*
